# Touch Research–Quo Vadis? A Plea for High-Quality Clinical Trials

**DOI:** 10.3390/brainsci11010025

**Published:** 2020-12-28

**Authors:** Bruno Müller-Oerlinghausen, Michael Eggart

**Affiliations:** 1 Charité Universitätsmedizin Berlin, 10117 Berlin, Germany; 2 Faculty Social Work, Health and Nursing, Ravensburg-Weingarten University of Applied Sciences, 88250 Weingarten, Germany; michael.eggart@rwu.de

Recently, the issue of a lack of interpersonal touch has gained much public interest due to the social distancing ordered by the authorities in the present pandemic situation [1]. Discussions on the social and medical value of touch nowadays will not be restricted to the realm of sociology, psychotherapy or philosophy but can refer to the ongoing and steadily increasing international research on the effects of touch in animals and humans and their underlying biological mechanisms [2]. It is largely to the credit of Swedish researchers that a new and fascinating branch of research has emerged within the last two decades, e.g., through the exploration of particular unmyelinated nerve fibers in the non-glabrous skin that specifically respond to gentle, interpersonal touch mediating feelings of well-being. From an epistemological point of view, it is noteworthy that the discovery of the so-called C tactile afferents (CT) in mammalians coincided with the emergence of social neuroscience in the early 1990s, probably marking a paradigm shift in the life sciences. In this respect, neurophysiologists have coined the term “affective touch”, which relates to both affection through skin-to-skin contact and positive affective states that are elicited by the touch experience [2]. Considering interpersonal touch from the bottom-up, the affiliative nature of affective touch is mainly, but probably not exclusively mediated by the CT system in the non-glabrous skin projecting to brain regions, which have been associated with social reward and interoception. However, a comprehensive understanding of social touch also includes top-down processes involving contextual factors that shape the touch experience, mainly represented by (a) the person giving touch (“who”) and (b) the underlying intention (“why”) [3]. Interestingly, the term “affective touch” was already used in the context of research into nursing in the 1980s and referred to touch experiences that are not task-related but can satisfy the basic human need for touch [4].

Many studies have explored the effects of various kinds of affective touch on the nervous system as well as on various neuropeptides such as oxytocin, stress hormones, the vagal tone, immunological parameters, etc. [5]. Recent studies have also investigated the neural processing of affective touch in mental disorders (see, e.g., [6] in this Special Issue). However, the introduction of specified touch techniques, e.g., psychoactive massage therapy into the treatment of psychiatric illness such as depression, anxiety or psychosomatic disorders as well as chronic pain, e.g., in patients with cancer [7], still meets much skepticism or open resistance often revealing stupendous ignorance of the existing scientific findings.

In the following, we are going to present some arguments why therapeutic touch should find its place within the multimodal therapy of particularly affective disorders. We shall particularly present our personal views on what kind of clinical studies in this area should be designed and performed in the future. This discussion will also touch on certain elements of the original articles collected in this Special Issue of *Brain Sciences*, which we had the pleasure and honor to edit.

Why do we need more and novel therapeutic approaches to the area of affective disorders? So far, the main treatments of depressed patients consist of either antidepressant drugs or psychotherapy. However, their general effectiveness is limited and far from satisfactory. This refers not only to the use of antidepressants, whose steadily increasing use and the spectrum of adverse drug reactions have provoked critical voices, but also to cognitive behavioral psychotherapy and mindfulness techniques. Their overall efficacy might also have been overestimated in the past [8,9,10,11,12]. Accordingly, it has been shown that one third of patients still present with residual symptoms at the end of treatment, which have been linked to worse long-term outcomes by predicting recurrent depression and significantly reducing quality of life [13]. Thus, an urgent need exists for the development of new and safe treatments for this indication, which are also accepted by the inflicted patients. It might be an interesting signal that particularly depressed patients and among them often women seek help from complementary treatments, and among them especially all kinds of massage [14]. In fact, some earlier and more recent systematic literature surveys including a meta-analysis of existing clinical studies strongly support the effectiveness of various kinds of massage in depressed patients in spite of various methodical deficits and particularly the heterogeneity of the studies performed so far [15,16,17].

What can be done in the future on behalf of academic medicine, clinical psychology, and basic research to make touch therapies attractive and an accepted treatment option for psychiatric or psychosomatic patients? We argue that a broader acceptance of touch in medicine will only be achieved on the basis of valid and high-quality scientific studies. There are, however, some important requirements, which should be met in future studies and scientific approaches. 

First of all, we are in need of excellent clinical studies comparing the mental and somatic effects and particularly anti-depressive efficacy of various kinds of affective touch, e.g., realized as psychoactive massage vs. established body-focused treatments such as relaxation methods, aerobic/anaerobic training, mind-body techniques, etc. It should be clarified whether and if so in which respect will the effects of massage using affective, gentle touch differ from those of classical (Swedish) massage in patients with affective or psychosomatic disorders. The applied manual techniques have to be described precisely (see, e.g., [18,19]), a postulate that has already been brought forward in various reviews [15,16]. Accurate description of the applied methods is also important, since some authors have argued that moderate pressure and not exclusively light stroking is required to obtain useful therapeutic effects. This argument has been brought forward particularly against the background of the hypothesis that it is the increased vagal tone, which explains the observed therapeutic effects, also, e.g., on the immune system [5]. Despite methodological limitations, another study has recently emphasized the crucial role of deep pressure touch for social bonding and potential clinical effects [20].

Standardized observer and self-rating scales for depression or the general state of well-being can and should be used to assess touch effects, although one should keep in mind that according to our own experience, patients after a potentially emotionally deeply “touching” massage might not feel extremely motivated to do a mass of paperwork. Visual analogue scales constructed individually on the basis of foregoing open trials and informal exchange with the patients about their reactions toward the applied treatment might often be the better choice to assess short-term effects [18,21]. Besides, there is the need to consider qualitative approaches in future research, e.g., by using (un)structured in-depth interviews aiming to gain insights into the subjective experience of patients and healthy volunteers. Although touch research has emphasized the psychological dimensions of affective touch by demonstrating positive effects on pleasantness and well-being assessed via standardized rating scales, there is a considerable lack of research elucidating the qualitative nature of these feelings in a differentiated manner. The preference for neuroscientific approaches over phenomenological research still dominates modern clinical psychiatry and may have limited the understanding of the subjective experiences that constitute the core of psychopathology [22]. Therefore, we argue for a consideration of phenomenological approaches that can be mixed with the quantitative paradigm to provide further insights into the “corporeality” [22] of mental disorders and the intervening function of touch therapies. A “qualitative turn” in touch research could also facilitate the generation of innovative hypotheses and might contribute to the development of a theory of human touch. From an epistemological point of view touch research should aim at not explaining the effects of touch in a purely reductionistic model being based on a single explanatory level and fostering a scientifically unsound mixing-up of psychological processes and brain functions. Instead, we argue for the exploration and discussion of the underlying mechanisms on separate explanatory levels.

A particularly tricky question in designing randomized-controlled trials in this scientific field is the choice of an adequate control condition. In view of the prevailing skepticism as to the usefulness of body-oriented treatments in psychiatric indications, it is essential to take care of a methodically conservative approach. Since “placebo controls” are difficult to be established, waiting lists or more or less boring “relax” videos have sometimes been used as controls that will provide the researcher with the prospect of more or less guaranteed positive effects of touch. Rapaport et al. recently presented a compelling research design that compared massage therapy with a light touch condition (laying on of hands) in patients with generalized anxiety disorders to identify effective touch techniques for managing clinical conditions [23]. The potential influences of, e.g., the appearance, age, sex (see, e.g., [24]) and empathy of the massage therapists and the special experimental environment should be reduced, e.g., by keeping the general experimental conditions as equal as possible in both groups. By giving special attention to this special methodical aspect, we might be able to isolate the effects of touch itself from other unspecific influences. There is a further need to establish equivalence studies comparing massage therapy’s efficacy with traditional approaches or standard care. Naturalistic studies will also provide more insight into the effectiveness of touch therapies in general settings.

Several mechanisms of action have been discussed, which could explain massage therapy’s clinical effects in patients suffering from affective disorders [16,25]. However, research to clarify these mechanisms is still in its infancy but could be conducive to the acceptance of touch therapies (see, e.g., [26]). The concept of interoception (i.e., “the sense of the physiological condition of the body” [27]) and its modification by various therapeutic approaches currently gains special attention. There is cumulative evidence for an association of various mental disorders with interoceptive impairments such as blunted heartbeat perception accuracy or maladaptive attention styles towards somatic feelings [28,29]. Interoceptive treatments are currently under development [30]; however, they focus mainly on mindfulness-based approaches [31], whose effectiveness in mental disorders may have been overestimated [32] and whose applicability to severely affected patients is limited [33,34]. Although affective touch offers an easy and safe access to the interoceptive system, the skin has so far received little attention as a modulator of interoceptive states. In a recent paper, we have proposed an interoceptive mechanism of action that could explain the well-documented anti-depressive effects of massage therapy by considering touch receptors in the skin that probably mediate the restoration of impaired interoceptive states [35]. To the best of our knowledge, this hypothesis has never been tested, although theoretical considerations support its fundamentals. Previous research has also validated instruments allowing the assessment of multidimensional self-reported interoception to identify interoceptive predictors of treatment outcome [36]. Moreover, another promising mechanism of massage therapy could be found in the promotion of restorative sleep patterns, which are probably associated with analgesic effects [37].

Touch may not be considered only as a therapeutic factor in psychiatry, since it also has a preventive function for mental health. This appears of particular importance in view of the detrimental effects of social isolation on morbidity and mortality in various cohorts [38]. An abundance of previous work has shown beneficial effects of gentle touch in the mother–infant dyad that have been identified as a resilience factor reducing the risk for later psychopathology and promoting attachment [39]. Baby massage has gained broad acceptance in many countries. However, only a small number of studies referred to the effects of massage on full-term newborns; stabilized sleep patterns, reduced bilirubin levels in jaundice and alleviating effects on maternal depression have been reported [40]. Evidence exists that depressed mothers give their babies less positive touch, which is likely to be compensated for by increased infant self-touching [41].

The applicability of affective touch in children as well as in elderly residents of nursing homes needs more attention and scientific approaches. Touch therapies and basal stimulation techniques could serve as a non-pharmacological strategy to deal with agitation in patients suffering from dementia [42]. Touch could also play a prominent role in patients who are cognitively unable to receive psychotherapy, such as individuals with severe intellectual disability [43]. As to psycho-oncology, some authors have contributed promising evidence on the supportive, analgesic, and relaxing effects of professional touch in cancer patients [44].

In summary, there is an urgent need for high-quality clinical trials examining and confirming the beneficial psychophysical effects of touch therapies in psychiatry. Further experimental research elucidating the mechanisms of action behind the mental effects of massage therapy certainly would also strengthen the acceptance of this intervention by public authorities, clinical medicine and the scientific community.
